# Are Metals Volatile?

**Published:** 1880-12

**Authors:** 


### ARE METALS VOLATILE?

Quite different views are held by chemists and practical smelters, and the question implies a waste or saving of about 10 per cent. of all the gold and silver smelted every year, amounting to fifty million dollars, thus an actual loss to the country of five million dollars annually by a needless waste, if metals are vaporous or become so under certain conditions of heat or chemical reactions, as held by Dr. Duryee.

Iron is entirely non-volatile, while the phosphorous in it is volatile if chlorine and sufficient heat be applied.

Lead is vaporized at a red heat; zinc about the same. The teluride of gold ores are found to be impossible of smelting in ordinary cupola furnaces, because telurium gives to gold the property of becoming volatile at a red heat, while gold itself is considered non-volatile at even a white heat; but it is found chlorine has the property of combining in a gaseous state with gold at a heat of four thousand degrees, rendering it so volatile that its vapors can be carried into a proper condenser and there condensed the same as oil vapors are condensed.

It is found by furnace men that silver and lead when smelted together are carried off to the extent of 10 to 30 per cent., even at ordinary furnace heat, and it is becoming a serious question around Leadville how to overcome the effect of lead poisoning from the numerous furnaces.

By several analyses of Duryee's furnace slags from gold and silver ores, it was found the metals were all vaporized down to ten to fifty cents per ton of ore, and as it is extremely easy and simple to condense these vapors, this mode of working the precious metals would seem to be the surest business sagacity on the part of smelting or mining companies.

It is stated semi-officially that Hill's Smelting Works will smelt a thousand tons a day at a profit of five million dollars a year even with a loss of 10 to 20 per cent. calculative loss by metals going off in vapor. Last year their profits were three million dollars. It is said the Grant furnaces at Leadville are earning five thousand dollars daily.

We repeat, it is sheer and unnecessary waste to lose even 10 per cent. by volatilization of metals, when by a proper condenser or horizontal cupola like Duryee's, they could be all saved, and lead poisoning vapors done away with. It now having been shown that all metals are vaporous, or can be made so, it is only a question of time when all these vapors will be saved. Who knows but that distilling of metals will be as easy as distilling oils or cider ! An oxy-hydrogen blow-pipe heat on a large scale properly applied would seem to us feasible and practical.

